# Sustained Improvement in Antibiotic Prescribing Among Private Practice Dentists Following Antibiotic Stewardship Education and Dashboard Implementation

**DOI:** 10.1093/ofid/ofag287

**Published:** 2026-05-18

**Authors:** Debra A Goff, Michael E Klesper, Kushal Dahal, Fnu Sampada, Minji Sohn, Jonathan Guerin, Jessica Allen, David Basali, Michael Edwards, Scott Ferguson, Krupa Patel, Fred Sakamoto, Jason Souyias, Nicholas Shumaker, Jackson Stoner, Jason Stoner, Douglas Goff

**Affiliations:** The Ohio State University Wexner Medical Center, Department of Pharmacy, Columbus, Ohio, USA; The Ohio State University College of Pharmacy, Columbus, Ohio, USA; Ferris State University College of Pharmacy, Big Rapids, Michigan, USA; Ferris State University College of Pharmacy, Big Rapids, Michigan, USA; Ferris State University College of Pharmacy, Big Rapids, Michigan, USA; Ferris State University College of Pharmacy, Big Rapids, Michigan, USA; Ferris State University College of Pharmacy, Big Rapids, Michigan, USA; Northern Colorado Periodontics, Fort Collins, Colorado, USA; Stoner Periodontics & Implant Specialists, Dublin, Ohio, USA; Implant Dentistry and Periodontics, Indianapolis, Illinois, USA; Scott Ferguson and Associates, Kimball, Michigan, USA; Periodontic Associates of Port Huron, Port Huron, Michigan, USA; Central Ohio Periodontics, Blacklick, Ohio, USA; Periodontic Associates of Port Huron, Port Huron, Michigan, USA; Northern Colorado Periodontics, Fort Collins, Colorado, USA; Dublin, Ohio, USA; Stoner Periodontics & Implant Specialists, Dublin, Ohio, USA; Dr's. Gilbert and Goff Prosthodontists, Columbus, Ohio, USA

**Keywords:** antimicrobial stewardship, behavior change, Current Dental Terminology, dentists, periodontist

## Abstract

**Background:**

The lack of procedure-specific, evidence-based dental antibiotic guidelines increases unnecessary and inappropriate antibiotic prescribing. The primary objective was to evaluate changes in antibiotic prescribing before and after antibiotic stewardship (AS) education by infectious diseases (ID) AS experts in addition to implementation of self-monitored antibiotic dashboards. A secondary objective was to link antibiotic prescriptions to standardized Current Dental Terminology (CDT) procedure codes.

**Methods:**

We conducted a retrospective, multi-practice cohort study among 9 private practice periodontists who completed AS education by ID-AS experts. Antibiotic data, CDT codes, and medical histories were extracted from electronic dental records from 1 January 2021 through 30 June 2025. Individualized dashboards displayed longitudinal antibiotic prescribing and linkage to CDT procedure codes. Outcomes were compared across pre-education, post-education year 1, and post-education year 3 periods.

**Results:**

A total of 11 795 antibiotic prescriptions for 7331 unique patients were identified; 85.6% were therapeutic (≥3 days) and 14.4% were prophylactic. Prophylactic antibiotics for patients with prosthetic joint implants decreased by 89% by post-education year 3 (*P* < .05). Clindamycin and fluoroquinolone use declined by 85% (*P* < .001) and 31% (*P* < .15), respectively. Ten-day antibiotic durations decreased by 87% (*P* < .001) with a corresponding increase in courses of 3–5 days across multiple CDT procedure categories. Improvements were sustained 3 years after education without ongoing external audit and feedback.

**Conclusions:**

Dentists demonstrated sustained improvements in antibiotic use following ID-AS–led stewardship education and implementation of self-monitored antibiotic dashboards. Linking prescriptions to CDT codes supports future development of evidence-based dental antibiotic guidelines.

## Teaser

Private practice periodontists significantly decreased antibiotic duration of therapy by 87% after antibiotic stewardship education by ID ASP experts and implementation of antibiotic dashboards. Linking CDT codes to antibiotic prescriptions allows for future development of evidence based antibiotic guidelines.

## Graphical Abstract

**Graphical Abstract:**
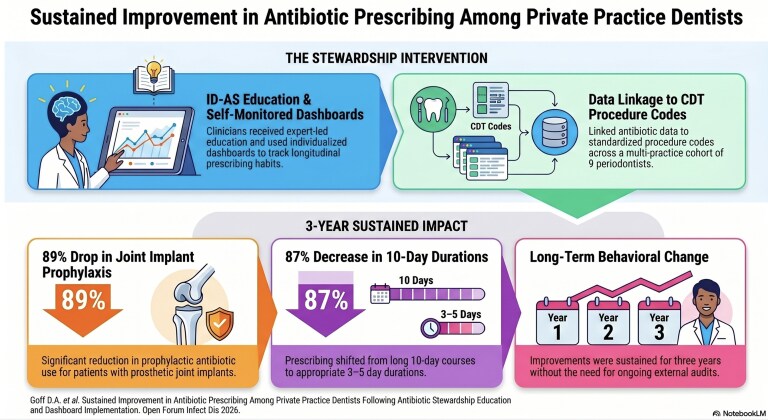

Private practice dentists represent approximately 72% of the dental workforce in the United States [1]. Dentists prescribe an estimated 25 million outpatient antibiotic courses annually [2]. Although general dentists account for most dental antibiotic volume, periodontists prescribe antibiotics at disproportionately higher rates per provider and contribute substantially to clindamycin use [2, 3]. Clindamycin is associated with a higher risk of *Clostridioides difficile* infection (CDI), making its use a key target for antimicrobial stewardship (AS) interventions [4]. Periodontists commonly prescribe antibiotics; however, the absence of evidence-based dental antibiotic guidelines increases the risk of unnecessary or inappropriate prescribing [5]. Prior analyses suggest that up to 80% of dental antibiotic prescriptions may be unnecessary, largely due to guideline gaps and limited implementation of AS principles [6].

Antibiotic decisions among dentists are strongly influenced by published guidelines and expert opinion [7]. Despite this reliance, only 2 American Dental Association (ADA) guidelines address antibiotic use in dentistry, and neither provides recommendations for dental implant procedures, a rapidly expanding procedure for both general dentists and specialists [8, 9]. More than 3 million individuals in the United States have dental implants, with an estimated 500 000 new implant placements annually [10]. As implant procedures increase, so does antibiotic exposure, underscoring the need for targeted dental AS interventions and collaboration with infectious diseases antibiotic stewardship (ID-AS) experts.

Although oral and systemic health is closely linked, medical and dental care delivery remain largely siloed. Limited interoperability between electronic health records (EHRs) and electronic dental records (EDRs) can compromise patient safety, particularly for antibiotic prescribing decisions that depend on accurate medical histories. Identification of patients at high risk for infective endocarditis is essential for appropriate prescribing [11]. In private practice settings, dentists rely on patient-reported histories and medical clearance forms rather than integrated EHR access. Unlike dental clinics embedded within health systems that use unified EHR platforms, private practice dental offices utilize a wide range of EDR software with variable data extraction capabilities. A prior dental AS study with private practice dentists demonstrated substantial challenges in obtaining structured antibiotic prescribing data and linking prescriptions to dental procedures [12].

Standardizing dental antibiotic assessment through linkage with Current Dental Terminology (CDT) procedure codes offers a potential solution. Current Dental Terminology codes are universally used for documentation and billing in dentistry and provide a structured framework to associate antibiotic prescribing with specific dental procedures. Large-scale datasets linking antibiotics to CDT codes for periodontal procedures could support the development of evidence-based antibiotic guidelines and enable benchmarking across practices.

The Collaborating to Harmonize Antimicrobial Registry Measures (CHARM) platform is an electronic antibiotic dashboard developed to support outpatient AS by enabling clinicians to review longitudinal prescribing patterns and assess concordance between antibiotic selection and documented indications [13]. Although CHARM has been used extensively in medical outpatient settings using International Classification of Diseases (ICD-10) codes, its application in private practice dental offices has not been previously described.

The purpose of this study was to implement CHARM antibiotic dashboards in private practice dental settings following antibiotic stewardship education delivered by ID-AS experts. The primary objective was to evaluate changes in antibiotic prescribing before and after stewardship education, in addition to implementation of self-monitored antibiotic dashboards. We hypothesized that the combination of expert-led education and longitudinal dashboard feedback would result in sustained improvements in dental antibiotic prescribing. A secondary objective was to link antibiotic prescriptions to CDT procedure codes for dental implants and other common periodontal procedures, providing a foundation for future evidence-based guideline development.

## METHODS

### Study Design and Oversight

This retrospective, multi-practice cohort study was conducted among private practice dental specialists between 1 January 2021 and 30 June 2025. Three prespecified study periods were evaluated for each participating dentist:

1. Pre-education period, defined as the 12 months preceding completion of dental AS education
2. Post-education year 1, defined as the first 12 months following education
3. Post-education year 3, defined as the 12-month period occurring 3 years after education

The study was approved by the Institutional Review Board of The Ohio State University. All dentists volunteered to participate.

### Patient Consent Statement

The study does not include factors necessitating patient consent.

### Study Population

Private practice periodontists participating in local dental education networks who attended a 3-hour dental ID-AS educational program between 2021 and 2022 were invited to participate. Periodontists were selected due to their high frequency of antibiotic prescribing for periodontal surgical procedures, including dental implant surgery, and the absence of updated evidence-based antibiotic guidelines from the ADA or the American Academy of Periodontology (AAP) specific to these procedures. The sample size of periodontists who volunteered to participate was determined by feasibility, available personnel, and funding.

### Antibiotic Stewardship Education

The dental AS education consisted of a structured, interactive 3-hour program delivered by ID-AS experts [12]. Content addressed 6 core domains: (1) evidence-based antibiotic prescribing; (2) risks of clindamycin and fluoroquinolone use, including CDI supported by a patient video and recommendations to document CDI histories in dental medical forms and use doxycycline or azithromycin; (3) shortening duration of therapy, informed from 162 medical trials due to limited dental data; (4) antibiotic prophylaxis for patients with prosthetic joint implants (PJI), emphasizing prescriber liability and appropriate responsibility for prescribing; (5) management of penicillin allergy, including referral for allergy evaluation and alternatives to clindamycin; and (6) interdisciplinary collaboration, supported by a sample communication letter to orthopedic surgeons. The program concluded with practical ways to implement these 6 domains of AS in a dental practice.

### Electronic Dental Record (EDR) Systems

There are over 100 EDR platforms used in private dental practices. We consulted with private practice dentists and used publicly available data to select 2 EDR systems, Dentrix and Open Dental. Dentrix is the market leader and has been available since 1989, whereas Open Dental, introduced in 2003, is an open-source platform with extensive customization capabilities.

### Study Definitions

Antibiotic prophylaxis was defined as a single antibiotic dose administered prior to a dental procedure. Therapeutic antibiotic use was defined as any post-procedure antibiotic prescription for ≥3 days, with or without an associated prophylactic dose.

### Data Sources and Eligibility Criteria

Eligible records included adult patients aged 18–95 years who received an oral antibiotic prescription for a dental procedure, either for prophylaxis or treatment. Patients prescribed antibiotics for non-dental indications and practices relying on paper chart were excluded.

All de-identified antibiotic prescriptions were extracted from EDRs and included prescription date, antibiotic agent, dose, dosing frequency, and quantity dispensed. Medical histories were abstracted, including reported allergies, presence of PJI, risk factors for infective endocarditis, history of *C. difficile* infection, and comorbid conditions associated with dental implant failure (smoking, vitamin D deficiency, osteoporosis, and diabetes mellitus).

### CDT Procedure Codes

Current Dental Terminology codes are maintained by the ADA and represent the national standard for documenting dental procedures. The CDT code set includes more than 845 codes across 13 categories and is updated annually. Because data were de-identified, it was not possible to reliably bundle multiple CDT codes to a single patient-level procedure. When more than one CDT code was recorded during a patient visit, it was not possible to determine whether the codes represented one procedure or multiple procedures performed on the same day. As a result, each CDT code was analyzed separately. For example, a single dental visit may include multiple CDT codes for services such as examination, anesthesia, implant placement, and bone grafting. In addition, many procedures were coded using “unspecified” codes when new materials or techniques had not yet been assigned a specific CDT code. All CDT codes associated with antibiotic prescriptions were extracted. Analysis therefore focused on the most frequently used CDT codes for periodontal procedures, including dental implants, scaling and root planing, and tooth extractions.

### Antibiotic Dashboard Development and Implementation

**Figure 1.**
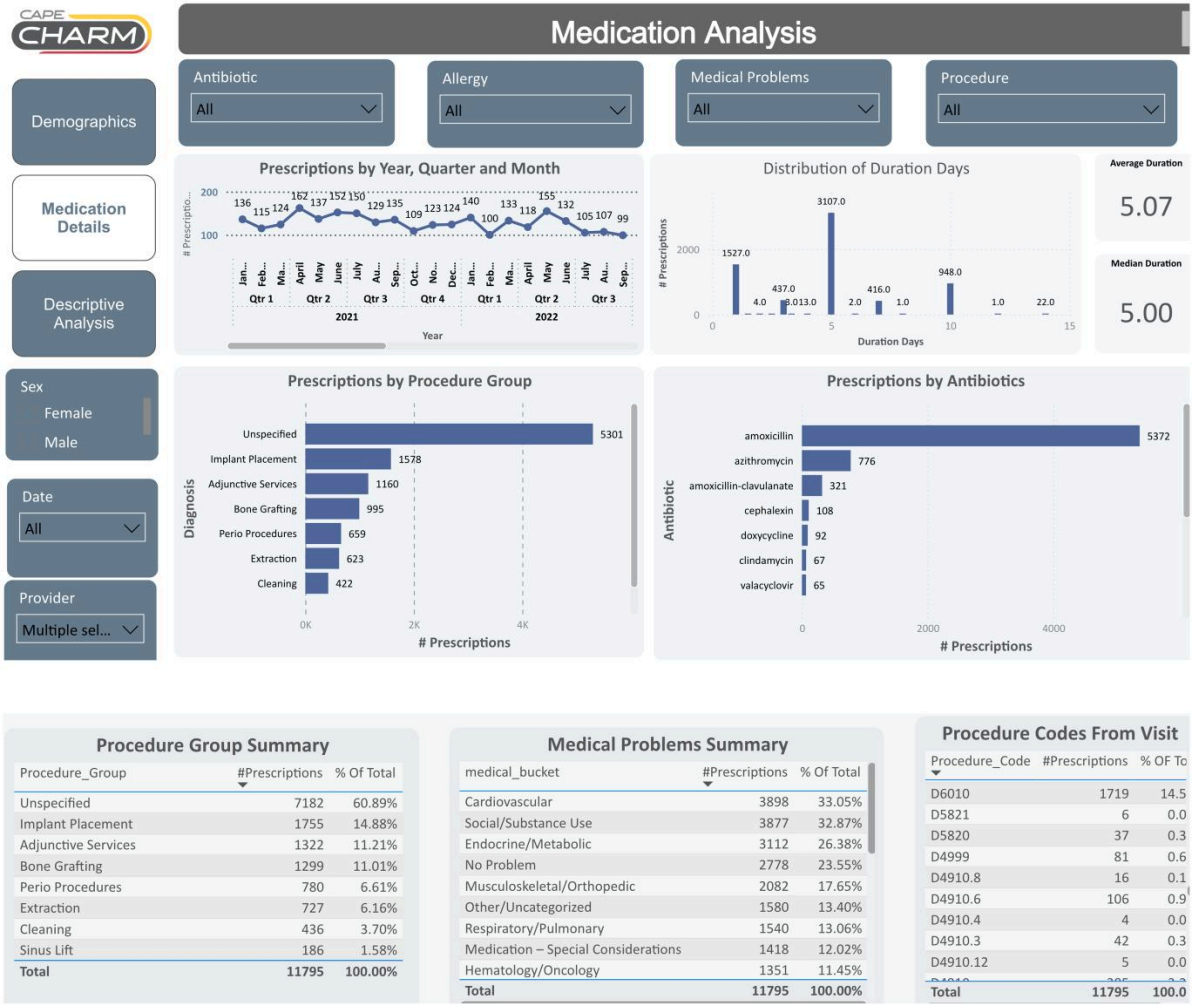
The CHARM (Collaborating to Harmonize Antimicrobial Registry Measures) antibiotic dashboard. Credit: Developed by Ferris State University College of Pharmacy.

Study faculty collaborated with one designated dental staff member from each participating practice to securely extract and transfer de-identified antibiotic prescribing data, CDT codes, and medical histories from Open Dental and Dentrix systems. For practices using Open Dental, structured data were extracted using structured query language queries within the Open Dental query editor connected to the centralized database. Dentrix lacked a native database query function; therefore, data were extracted manually using standardized reports generated directly from the Dentrix system. This required additional time by IT experts and multiple discussions with the dentists. Data from 2 dentists were excluded from the duration-of-therapy analysis due to incomplete longitudinal data and EDR default antibiotic duration settings that did not reflect actual prescribing intent which could have resulted in misclassification. One dentist practiced across multiple offices; however, data extraction from the primary office identified only 2 antibiotic prescriptions over 4.5 years. Because the dental software was not networked across locations and additional data extraction was not feasible due to limited IT resources, this dentist was excluded. All data were consolidated into a secure relational database and standardized for analysis. Individualized antibiotic dashboards were developed using Microsoft Power BI and displayed longitudinal prescribing patterns, antibiotic selection, duration of therapy, prophylactic versus therapeutic use, medical history, and linkage to CDT procedure codes (Figure 1).

### Dashboard Review and Qualitative Feedback

Dashboards were introduced in 2025 using principles of implementation science and behavior change theory. Dentists received a secure, dashboard link and participated in a one-on-one review session with the study principal investigator. Dentists were guided in navigating each section to encourage self-reflection and promote sustained behavior change based on 4.5 years of individual antibiotic prescribing patterns. The dashboard link allowed unlimited views. Dashboards were not updated monthly as this required additional IT support. Dentists provided qualitative feedback following the session.

### Statistical Analysis

Statistical analyses were performed using SAS version 9.3 (SAS Institute, Cary, NC). Categorical variables were compared using the χ² test or Fisher’s exact test, as appropriate. Continuous variables were analyzed using Student's t-test. All tests were 2-sided, and a *P* ≤ .05 was considered statistically significant. Comparisons of antibiotic prescription counts across equal observation periods were additionally assessed using exact Poisson tests.

## RESULTS

Nine periodontists prescribed 11 795 antibiotic prescriptions for 7331 unique patients over the 4.5-year study period. The final analytic cohort for duration of therapy included 6922 antibiotic prescriptions written for 4515 unique patients.

### Antibiotic Prescribing Patterns

Overall, 14.4% of antibiotic prescriptions (n = 1693) were for prophylaxis, while 85.6% (n = 10 102) were therapeutic prescriptions of ≥3 days’ duration. Among prophylactic prescriptions, 108 were written for patients with documented risk factors for infective endocarditis, including artificial heart valves (n = 86), prior infective endocarditis (n = 9), heart transplant (n = 2), and congenital heart disease (n = 11). A total of 321 prophylactic antibiotic prescriptions were written for patients with PJI. Following stewardship education, prophylactic antibiotic prescribing for PJI patients decreased by 89% (*P* < .05) from 117 prescriptions pre- to post-education year 1 (n = 110), year 2 (n = 82), and year 3 (n = 12). Most prophylactic prescriptions (n = 1264) were prescribed for dental surgery.

The most frequently prescribed antibiotics are shown in Table 1. Amoxicillin accounted for 71.6% of prescriptions (n = 8453), followed by doxycycline (10.0%), azithromycin (7.8%), amoxicillin-clavulanate (6.8%), cephalexin (0.9%), and clindamycin (0.6%). Pre- and post-education year 3, clindamycin use decreased by 85% (*P* < .001) and quinolone use declined by 31% (*P* = .153).

**Table 1.** Antibiotic Prescriptions Pre-education and Post-education 1 Year and 3 Years

	Rx Over 4.5 years, Percent (n)	Pre-education (2021), Percent (n)	Post-education, 1 year (2022), Percent (n)	Post-education, 3 year (2024), Percent (n)	*P*-Value^a^
Total Rx^b^	11 795	2388	2383	2850	
Amoxicillin	72% (8453)	79% (1879)	74% (1757)	69% (1956)	
Doxycycline^c^	10% (1169)	5% (121)	10% (243)	11% (318)	
Azithromycin	8% (921)	8% (180)	8% (201)	8% (220)	
Amoxicillin-clavulanate	7% (800)	3% (82)	4% (104)	9% (249)	
Cephalexin	0.95% (112)	1% (22)	1% (26)	1% (31)	
Clindamycin	0.65% (77)	2% (54)	0% (0)	0.2% (8)	<.001
Quinolones	0.40% (47)	0.67% (16)	0.21% (5)	0.39% (11)	.153
Cefdinir	0.29% (34)	0.04% (1)	0% (0)	0.25% (7)	
Metronidazole	0.21% (25)	0.25% (6)	0.25% (6)	0.14% (4)	

^a^ *P* value calculated on pre- (2021) and post-education year 3 (2024).

^b^Rx = prescriptions.

^c^30-day sub-antimicrobial dose doxycycline (Periostat®) prescriptions were excluded from the analysis.

Penicillin allergy was reported in 12% of patients (n = 890) and accounted for 14% of all antibiotic prescriptions (n = 1639). Prior to stewardship education, azithromycin (47%, n = 127) was the most prescribed agent among patients reporting penicillin allergy, followed by clindamycin (14%, n = 38) and doxycycline (13%, n = 36). At 1-year post-education, no clindamycin prescriptions were recorded in this group. By post-education year 3, doxycycline (40%, n = 175) and azithromycin (37%, n = 165) remained the most frequently prescribed agents, with minimal use of cephalexin (1%, n = 6) and clindamycin (1%, n = 6).

In addition to infections, other risk factors for dental implant failure were identified: smoking (n = 1738, 14.7%), diabetes mellitus (n = 1068, 9%), osteoporosis (n = 839, 7%), and vitamin D deficiency (n = 17, 1%). A history of CDI was identified in 17 patients.

### CDT Procedure Codes and Duration of Therapy

Current Dental Terminology procedure codes associated with antibiotic prescriptions are summarized in Table 2. Comparisons of antibiotic duration of therapy across pre-education, post-education year 1, and post-education year 3 periods demonstrated a significant (*P* < .001) and consistent shift from traditional 10-day durations to shorter durations across all evaluated procedure categories. Ten-day durations decreased by 82% for tooth extractions, 97% for scaling and root planing, 85% for surgical implant placement, 85% for sinus lift dental implant procedure, and 71% for implant removal.

**Table 2.** Current Dental Terminology (CDT) Codes and Antibiotic Duration of Therapy Pre- and Post-education 1 Year and 3 Years

Duration of Therapy For Summed CDT Codes	Duration of Therapy (Days)	Pre-education (2021), n = 1006	Post-education, 1 Year (2022), n = 750	Post-education, 3 Years (2024), n = 1127	*P*-Value^a^
	10	363	104	46	*P* = <.001
	7	48	22	117	*P* = <.001
	5	252	363	457	*P* = <.001
	3	58	66	175	*P* = <.001
	1	285	195	332	*P* = .566

Dental Implant Codes	Duration of Therapy (Days)	Pre-education (2021), n, Percent^b^	Post-education, 1 Year (2022), n, Percent	Post-education, 3 Years (2024), n, Percent
D6010, surgical implant placement		n = 445	n = 310	n = 350
	10	140 (31%)	48 (15%)	21 (6%)
	7	13 (3%)	7 (2.3%)	94 (1%)
	5	92 (21%)	133 (43%)	128 (37%)
	3	23 (5%)	36 (12%)	111 (7%)
	1	157 (35%)	81 (26%)	164 (47%)
D4273, bone grafting, autogenous graft		n = 57	n = 39	n = 56
	10	26 (46%)	10 (26%)	4 (7%)
	7	2 (4%)	1 (3%)	2 (4%)
	5	24 (42%)	27 (69%)	46 (82%)
	3	0	1 (3%)	3 (5%)
	1	0	0	1 (2%)
D6104, bone graft at time of implant placement		n = 13	n = 16	n = 30
	10	2 (15%)	1 (6%)	1 (3%)
	7	0	1 (6%)	0
	5	7 (54%)	12 (75%)	29 (97%)
	3	0	0	0
	1	4 (31%)	2 (12%)	0
D7953, bone replacement graft after tooth extraction		n = 42	n = 31	n = 35
	10	18 (43%)	10 (32%)	1 (3%)
	7	0	0	5 (14%)
	5	12 (29%)	17 (55%)	16 (46%)
	3	0	0	1 (3%)
	1	10 (24%)	3 (10%)	12 (34%)
D7950, bone grafting autogenous or nonautogenous		n = 54	n = 28	n = 18
	10	21 (39%)	5 (18%)	1 (6%)
	7	6 (11%)	0	0
	5	13 (24%)	7 (25%)	7 (39%)
	3	6 (11%)	8 (29%)	4 (22%)
	1	6 (11%)	8 (29%)	6 (33%)
D7951, sinus lift lateral approach		n = 23	n = 19	n = 40
	10	10 (43%)	0	2 (5%)
	7	0	3 (16%)	0
	5	3 (13%)	12 (63%)	20 (50%)
	3	0	3 (16%)	7 (18%)
	1	10 (43%)	1 (5%)	11 (28%)
D6100, 6100.1, 6100.2, surgical removal of dental implant		n = 43	n = 15	n = 27
	10	22 (51%)	4 (27%)	0
	7	1 (2%)	0	0
	5	10 (23%)	2 (13%)	24 (89%)
	3	2 (5%)	1 (7%)	2 (8%)
	1	6 (14%)	1 (7%)	1 (4%)
D4341, D4342, scaling and root planing		n = 85	n = 58	n = 62
	10	40 (47%)	4 (7%)	1 (2%)
	7	9 (11%)	3 (5%)	0
	5	22 (26%)	42 (72%)	42 (68%)
	3	6 (7%)	6 (10%)	5 (8%)
	1	7 (8%)	3 (5%)	14 (23%)
D7210, D 7140, tooth extraction		n = 276	n = 261	n = 341
	10	84 (30%)	22 (9%)	15 (4%)
	7	17 (6%)	7 (3%)	16 (5%)
	5	69 (25%)	111 (43%)	145 (43%)
	3	21 (8%)	11 (4%)	42 (12%)
	1	85 (31%)	96 (37%)	123 (36%)

^a^ *P* value is calculated based on cumulative durations of therapies using pre-education (2021) and post-education year 3 (2024) across all CDT codes.

^b^Percents do not add up to 100% due to missing values and removal of 30-day durations of sub-antimicrobial dose doxycycline Periostat®.

### CHARM Dashboard Feedback

Each dentist completed one individualized antibiotic dashboard review session with the study principal investigator. Dentists consistently reported that this was their first opportunity to review their own antibiotic prescribing patterns longitudinally over a 4.5-year period. Qualitative feedback regarding usability and perceived value of the dashboards was uniformly positive. Representative verbatim comments are presented in Table 3.

**Table 3.** Qualitative Feedback From Dentists Using CHARM Antibiotic Dashboard

I stopped prescribing clindamycin and I see it is reflected in my dashboard.
I changed my duration of therapy from 10 days to 5 days for most procedures. Those few post-education 10-day prescriptions must be for my patients who are not able to see me immediately because they are out of town.
I did not think the data was accurate. I changed my standard duration to 5-days post-education but upon further review, I found I had pre-set antibiotic template defaults still set at 10 days.
It was eye opening to see 4.5 years of my prescribing. I still see some 7-10 day duration post education. I need to investigate this.
We need to do better due diligence with the medical history. Post-education I changed my medical form to list the 4 risk factors for infective endocarditis and a history of *C. difficile* infections. The dashboard lets me see the medical histories associated with my prescribing. It would be great if medical and dental electronic records talked to each other.
I have never seen my own antibiotic prescribing. This is really cool to see how I changed my use of clindamycin and shortened my durations over 4.5 years. It would be great to get a monthly dashboard update.
This is helpful to learn how many of my patients have risk factors for dental implant failure. I have never seen medical histories and antibiotic prescriptions linked to specific CDT codes. I think this data can help us come to a consensus on what is the appropriate use of antibiotics for perio procedures.
I love it! This is so helpful to me. I am glad to see my prescribing aligns with the six stewardship concepts I learned.
The dashboard is very simple to navigate. I like looking at my outlier prescriptions with 7-10 day durations to see what CDT codes they are associated with.

## DISCUSSION

To our knowledge, this is the first US multi-practice antibiotic stewardship intervention study with private practice dentists that demonstrated sustained and clinically meaningful improvements in antibiotic prescribing among periodontists following education by ID-AS experts and implementation of self-monitored CHARM antibiotic dashboards. Most notably, dentists decreased clindamycin use and shifted their standard duration of postoperative antibiotic therapy from traditional 7- and 10-day courses to 3- and 5-day regimens across multiple periodontal and dental implant-related CDT procedure codes. This change was observed at both 1 year and 3 years post-education, supporting the durability of behavior change when education is paired with longitudinal prescribing feedback.

### Shortening Duration of Therapy

The reduction in antibiotic duration represents a critical stewardship outcome. Historically, prolonged courses of antibiotics in dentistry have been driven by dental school teaching, expert opinion, anecdotal experience, and concern for postoperative complications, rather than robust evidence. In this study, 10-day antibiotic courses decreased by 87% with a corresponding increase in courses of 3–5 days. These findings align with broader AS literature in medicine demonstrating that shorter courses are equally effective for many infections [14]. Although US evidence-based dental implant antibiotic duration guidelines remain limited to dental experts [15, 16], the observed prescribing shifts reflect increasing alignment with emerging data and stewardship principles favoring the shortest effective duration.

### Role of Antibiotic Dashboards and Implementation Insights

A key strength of this intervention was the use of individualized antibiotic dashboards, which allowed dentists to visualize their prescribing patterns over a 4.5-year period. Importantly, dashboard review identified a critical implementation issue that would not have been detected through self-report alone: one dentist's standard 10-day antibiotic duration did not change post-education because preset EDR templates, configured years earlier, remained unchanged. As a result, these data would have been misclassified as non-compliant with AS recommendations due to a preset system-level template, rather than reflecting the prescriber's intent. This finding underscores the importance of pairing education with objective data and highlights how EDR defaults and templates can inadvertently perpetuate inappropriate prescribing. A study done in a large health system [17] also found that default 10-day antibiotic durations embedded in electronic prescription order entry led to inappropriately long antibiotic courses and unnecessary days of therapy. After default removal, prescriptions with a 10-day duration declined. The CHARM dashboard not only facilitated accurate measurement of prescribing behavior but also functioned as a diagnostic tool to identify system-level barriers to change. Compared with traditional audit-and-feedback approaches which are resource-intensive and require sustained external oversight by ID-AS experts, self-monitored dashboards offer a scalable strategy for private practice settings.

In a prior private practice dental AS study using audit-and-feedback methods, dentists demonstrated fixed prescribing patterns with preferred standard durations of 5, 7, 10, or 14 days, largely independent of procedure type [12]. Using weekly audit and feedback by ID-AS experts, mean durations decreased from 7.7 days pre-intervention to 5.1 days post-intervention (*P* < .0001). Longer durations (10–14 days) were most frequently associated with dental implants. Our study used AS education with implementation of CHARM antibiotic dashboards. This was associated with immediate and sustained 3-year reductions in antibiotic duration across multiple CDT procedure categories (Table 2) without time-intensive weekly audit and feedback.

Dentists described frustration with having to rely on patient recall for medical histories instead of accessing physician-documented data within the medical EHR. This lack of interoperability between medical EHRs and dental electronic records was recently addressed by Patel et al [18].

### Reduction in Prophylactic Antibiotics for PJI

Another important finding was a statistically significant 89% reduction in prophylactic antibiotic prescribing among patients with PJI following stewardship education. This gradual reduction suggests that targeted education by ID-AS experts can effectively address persistent misconceptions and reduce unnecessary antibiotic use without compromising patient safety. ID-AS experts emphasized, consistent with ADA guidance, that when orthopedic surgeons recommend dental antibiotic prophylaxis, the responsibility for prescribing should reside with the recommending surgeon. Importantly, liability for adverse outcomes related to unnecessary antibiotic use rests with the prescribing clinician, a factor that has been shown to drive defensive antibiotic prescribing among both dentists and orthopedic surgeons [19]. Unnecessary prophylaxis remains common nationally, fueled by medicolegal concerns and inconsistent cross-disciplinary messaging [2]. In 2024, the American Association of Orthopedic Surgeons published updated evidence-based guidelines [20] stating that routine dental antibiotic prophylaxis for patients with PJI is no longer indicated, while acknowledging anticipated challenges in guideline adoption by orthopedic surgeons. These findings highlight an important opportunity for dentists to apply AS principles and engage in collaborative, evidence-based care by informing orthopedic colleagues and patients of updated recommendations.

### Decreased Clindamycin Use and Comparison With Prior Studies

The 85% reduction in clindamycin prescribing is particularly noteworthy. Data consistently show that dentists contribute disproportionately to clindamycin use, despite its association with CDI and lack of superiority for most odontogenic infections [21]. Similar reductions in clindamycin have been reported in prior dental stewardship studies; however, most report short-term outcomes [12, 22]. The sustained decrease observed here over several years suggests that combining education with self-directed feedback may be more effective than episodic interventions alone. The concurrent decrease in fluoroquinolone use, a drug class carrying four serious adverse event black-box warnings, further underscores improvement in antibiotic selection aligned with stewardship best practices.

### Dentists’ Application of Antibiotic Stewardship

The periodontists in this study are members of the Seattle Study Club, an international network of more than 7000 private practice dentists dedicated to interdisciplinary, comprehensive care. Local study clubs meet regularly and convene annually at a national meeting. The network's mission is to elevate the standard of patient care through ongoing clinical excellence. Dentists who participated in this study, together with the ID-AS expert involved in this study, provide ongoing AS education to Seattle Study Club members and dental colleagues. We encourage healthcare providers with expertise in outpatient AS to engage with academic and private practice dentists and dental hygienists. In addition, collaboration with established dental research networks, such as the Big Mouth Dental Research Group, the National Dental Practice-Based Research Network, and the US Department of Veterans Affairs could be beneficial for future studies [22, 23].

## LIMITATIONS

This study has limitations. First, the cohort was small and limited to periodontists, which may restrict generalizability to other dental specialties. Data extraction challenges varied by EDR platform, resulting in exclusion of 2 dentists from the duration-of-therapy analysis; implementation was more feasible among practices using Open Dental than Dentrix, which required substantial IT resources. Although CDT codes were linked to antibiotic prescriptions, de-identified data limited the ability to bundle multiple CDT codes into single procedures, reducing analytic granularity and inconsistent CDT documentation may have led to undercounting. Because the intervention included both education and dashboards, their independent effects could not be assessed; however, prescribing improvements occurred after education and before dashboard implementation. Dashboards identified preset templates that were corrected and further supported improved prescribing. CHARM dashboards were built with free Microsoft Power BI software; however, the implementation required IT expertise. Lastly, voluntary participation may have introduced selection bias.

## CONCLUSION

This study demonstrates that private practice dentists achieved sustained and clinically meaningful improvements in antibiotic prescribing, most notably through reductions in duration of therapy and clindamycin use following ID-AS education and implementation of antibiotic dashboards. This approach is less resource-intensive than traditional audit and feedback and scalable to broader settings. Future efforts should expand this model to additional dentists and leverage CDT-linked prescribing data to support development of evidence-based dental antibiotic guidelines, particularly for dental implants. Continued collaboration between dentists, infectious diseases antibiotic stewardship experts, orthopedic surgeons, and dental research networks will be essential to advance safe, effective, and evidence-based antibiotic use in dentistry.

## References

[1] American Dental Association Health Policy Institute . Practice ownership among dentists continues to decline. Available at: https://www.ada.org/-/media/project/ada-organization/ada/ada-org/files/resources/research/hpi/hpigraphic_practice_ownership_among_dentists_decline.pdf. Accessed 15 February 2026.

[2] Suda KJ, Calip GS, Zhou J, et al Assessment of the appropriateness of antibiotic prescriptions for infection prophylaxis before dental procedures. JAMA Netw Open 2019; 2:e193909. 31150071 10.1001/jamanetworkopen.2019.3909 PMC6547109

[3] Suda KJ, Roberts RM, Hunkler RJ, Taylor Jr TH. Antibiotic prescriptions in the community by type of provider in the United States, 2005–2010. J Am Pharm Assoc 2016; 56:621–6. 10.1016/j.japh.2016.08.015 27745794

[4] Brown KA, Khanafer N, Daneman N, Fisman DN. Meta-analysis of antibiotics and the risk of community-associated clostridioides difficile infection. Antimicrob Agents Chemother 2013; 57:2326–32. 23478961 10.1128/AAC.02176-12 PMC3632900

[5] Lockhart PB, Tampi MP, Abt E, et al Evidence-based clinical practice guideline on antibiotic use for the urgent management of pulpal- and periapical-related dental pain and intraoral swelling. J Am Dent Assoc 2019; 150:906–21. 31668170 10.1016/j.adaj.2019.08.020 PMC8270006

[6] Fleming-Dutra KE, Hersh AL, Shapiro DJ, et al Prevalence of inappropriate antibiotic prescriptions among US ambulatory care visits, 2010–2011. JAMA 2016; 315:1864–73. 27139059 10.1001/jama.2016.4151

[7] Palmer NO, Martin MV, Pealing R, et al Antibiotic prescribing knowledge of national health service general dental practitioners in England and Scotland. J Antimicrob Chemother 2001; 47:233–7. 11157915 10.1093/jac/47.2.233

[8] Sollecito TP, Abt E, Lockhart PB, et al The use of prophylactic antibiotics prior to dental procedures in patients with prosthetic joints. J Am Dent Assoc 2015; 146:11–6. 25569493 10.1016/j.adaj.2014.11.012

[9] American Dental Association . Chairside guide: antibiotic use for the urgent management of dental pain and intraoral swelling. Chicago, IL: American Dental Association; 2019. Available at: https://www.ada.org/resources/research/science/evidence-based-dental-research/antibiotics-for-dental-pain-and-swelling. Accessed 15 February 2026.

[10] American Academy of Implant Dentistry . Dental implant facts and figures. Available at: https://www.aaid.com. Accessed 20 February 2026.

[11] Wilson W, Gewitz M, Lockhart P, et al Prevention of Viridans group streptococcal infective endocarditis: a scientific statement from the American Heart Association. Circulation 2021; 143:e963–78. 33853363 10.1161/CIR.0000000000000969

[12] Goff DA, Mangino JE, Trolli E, Scheetz R, Goff D. Private practice dentists improve antibiotic use after dental antibiotic stewardship education from infectious diseases experts. Open Forum Infect Dis 2022; 9:1–9. 10.1093/ofid/ofac361 PMC9361170 35959211

[13] T B, Dahal K, Klepser ME, et al Collaboration to harmonize antimicrobial registry measures (CHARM) database analysis of antibiotic prescribing in urgent and non-urgent care: a retrospective study on demographic factors. Antimicrob Steward Healthc Epidemiol 2025; 5:e303. 41306705 10.1017/ash.2025.10197 PMC12645235

[14] Spellberg B, Rice LB. Duration of antibiotic therapy: shorter is better. Clin Microbiol Infect 2023; 29:145–7. 10.1016/j.cmi.2022.04.005 35436612

[15] Resnik R . Prophylactic antibiotic use in implant dentistry. Integrate 2023; 1:1.

[16] Rutkowski JL . Antibiotic considerations in implant dentistry. J Oral Implantol 2023; 49:221–8. 10.1563/AAID-JOI-D-4901.Editorial 36913697

[17] Wrenn RH, Diez T, Turner N, et al Duration of antimicrobial therapy: the impact of defaults. Open Forum Infect Dis 2018; 5:S85.

[18] Patel JS, Dinh E. LinkMD: linking medical and dental records with 4 linking algorithms. J Dent Res 2026; 105:16–20. 41254963 10.1177/00220345251383863

[19] Goff DA, Mangino J, Glassman A, Goff D, Larsen P, Scheetz R. review of guidelines for dental antibiotic prophylaxis for prevention of endocarditis and prosthetic joint infections and need for dental stewardship. Clin Infect Dis 2020; 71:455–62. 31728507 10.1093/cid/ciz1118

[20] American Academy of Orthopaedic Surgeons . Prevention of periprosthetic joint infection in patients undergoing dental procedures: evidence-based clinical practice guideline. Rosemont, IL: AAOS; 2024. Available at: https://www.aaos.org. Accessed 3 February 2026.

[21] Teoh L, Stewart K, Marino RJ, McCullough MJ. Antibiotic resistance and the role of dentists in prescribing. Antibiotics 2021; 10:673.34199987

[22] Gross AE, Hanna D, Rowan SA, et al Successful antimicrobial stewardship intervention in dental clinics. Open Forum Infect Dis 2019; 6:ofz067. 30895206 10.1093/ofid/ofz067 PMC6419992

[23] Walji M, Spallek H, Kookai K, et al BigMouth: development and maintenance of a successful dental data repository. J Am Med Inform Assoc 2022; 29:701–70. 35066586 10.1093/jamia/ocac001 PMC8922177

